# Multivariate risk prediction tools including MRI for individualized biopsy decision in prostate cancer diagnosis: current status and future directions

**DOI:** 10.1007/s00345-019-02707-9

**Published:** 2019-03-13

**Authors:** Ivo G. Schoots, Monique J. Roobol

**Affiliations:** 1 Department of Radiology and Nuclear Medicine, Erasmus MC University Medical Center, P.O. Box 2040, ‘s-Gravendijkwal 230, 3000 CA Rotterdam, The Netherlands; 2 Department of Urology, Erasmus MC University Medical Center, Rotterdam, The Netherlands

**Keywords:** Prostate cancer (PCa), Biopsy, Magnetic resonance imaging (MRI), MRI-guided targeted biopsy, PI-RADS, Risk stratification

## Abstract

**Background and purpose:**

Individualized risk-adapted algorithms in prostate cancer (PCa) diagnosis using predictive prebiopsy variables in addition to prostate-specific antigen value may result in a considerable reduction of unnecessary systematic biopsies. Multi-parametric magnetic resonance imaging (mpMRI) has emerged as a secondary prediction tool that can further improve the detection of clinically significant prostate cancer (csPCa). This review explores the performance of new MRI risk models for indicating a biopsy for prostate cancer diagnosis.

**Results and considerations:**

The area under the receiver-operating characteristic curve for detecting csPCa varies between 0.64 and 0.91 in biopsy-naïve men, and between 0.78 and 0.93 in men with a previous negative biopsy. The utility of multivariate risk prediction tools including MRI suspicion scores as an extra input parameter has the potential to avoid a notable number of biopsies and detection of clinically insignificant PCa at a low price of missing some csPCa. The trade-off depends on the risk threshold that is chosen. In biopsy-naïve men a net benefit was obtained at a risk threshold of above 10% for csPCa in most MRI risk prediction models. All constructed MRI risk models used (referral) patient cohorts with high prevalence of csPCa. Using more representative cohorts from daily clinical screening, net benefit may attenuate at lower risk thresholds. Strengths and limitations of these models are discussed.

**Future directions:**

To assess their wider applicability, in-depth analysis of mpMRI predictive qualities should be further investigated, in combination with required external validation of these models in a multicenter setting with large prospective datasets.

## Introduction

Transrectal ultrasound-guided systematic biopsy in the work-up of prostate cancer (PCa) diagnosis has shown a rising prevalence of antibiotic-resistant bacterial infections with biopsy-related septic complications [1]. Furthermore, systematic biopsy is associated with increased detection of indolent or low-grade PCa [2]. Reduction of systematic biopsies is pivotal in men who eventually prove to have no or low-grade PCa. Utility of validated multivariate risk prediction models to determine the risk of clinically significant PCa (csPCa) and to reduce (unnecessary) biopsy is nowadays recommended in guidelines [3].

The individualized risk-adapted approach in prostate cancer diagnosis is about to change with the introduction of prostate multi-parametric magnetic resonance imaging (mpMRI) in daily practice. Despite the qualities of mpMRI in predicting the absence or presence of csPCa, today mpMRI is still utilized as a diagnostic test for improving the performance of the diagnostic work-up, and not reducing biopsy [3, 4]. In other words, mpMRI is indicated when systematic biopsy is indicated, and thus when the likelihood is high of having clinically significant disease in a subsequently systematic biopsy.

mpMRI is especially indicated in men with prior negative biopsy who are still suspected of having significant disease. However, in biopsy-naïve men mpMRI is also suggested as an upfront or prebiopsy diagnostic test, to improve the diagnostic yield when combining targeted and systematic biopsy [5–7]. Moreover, mpMRI has also been introduced as a triage test to indicate performing or not performing a biopsy [5, 8–10]. As a result of its high negative predictive value, men with no suspected evidence for csPCa on MRI may defer systematic biopsy [11, 12].

Utilizing mpMRI as a triage test shows resemblance with risk prediction, and may have overlap with current multivariate risk prediction models for prostate cancer [13–19]. Most of these current risk models have been externally validated several times, and all use prostate-specific antigen (PSA), and digital rectal examination (DRE) as individual predictive input variables. To improve their predictive value, some use extra input variables such as age, prostate volume, free PSA, family history, race, and prior negative biopsy.

Due to the predictive value of mpMRI in PCa diagnosis, recently new multivariate risk prediction tools have been constructed, with the inclusion of the mpMRI suspicion score [20–29]. The purpose of this review is to explore the performance of these new MRI risk models for indicating a biopsy for prostate cancer diagnosis.

## Multivariate risk prediction including mpMRI for indicating targeted biopsy

Including the mpMRI as an extra diagnostic test in multivariate risk prediction tools, the balance between benefit and risks may change and should be (re-)evaluated. In this evolution, the first studies are published, mainly focusing on maximizing diagnostic yield, in combinations with potentially reducing biopsies and reducing the detection of clinically insignificant PCa. Study characteristics are shown in Tables 1 and 2. Theses multivariate prediction tools are based on logistic regression models. For these models, mostly the area under the receiver-operating-characteristic (ROC) curve (AUC) are investigated for any PCa and csPCa (Table 2), sometimes in combination with decision and net reduction curve analysis. In most studies clinically significant PCa was defined as Gleason score 3 + 4 or higher, nowadays better referred to the International Society of Urological Pathology (ISUP) grade (G) 2 and higher.

**Table 1:** Characteristics of studies related to nomogram development for prostate cancer diagnosis, including multi-parametric MRI

Study		Diagnostic work-up										
		Patient recruitment			Diagnostic protocol					Outcome measurement		
Patient groups	Year	Number of centers	Inclusion period	Criteria	Number of men	mpMRI/bpMRI	Positive	Targeted biopsy	Systematic biopsy	All PCa	csPCa	Definition csPCa
**Biopsy-naive setting**												
Alberts et al. [20]	2018	5	2012–2017	+PSA ± DRE	504	mpMRI	P3-5	Fusion	12-cores TR	y	y	GS ≥ 3 + 4
Bjurlin et al. [24] (Urology)	2018	1	Jun 2012–Aug 2014	+PSA ± DRE	201	mpMRI	P3-5	Fusion	12-cores TR	y	y	GS ≥ 7
		1	Jun 2012–Aug 2014		87							
Fang et al. [23]	2016	1	Jan 2011–Nov 2013	+PSA ± DRE ± TRUS	894	mpMRI	n.r.	Cognitive	12-cores TR	y	y	GS ≥ 4 + 3
Mehralivand et al. [22]	2018	1	May 2015- Aug 2016	+PSA ± DRE	400	mpMRI	P3-5	Fusion	12-cores TR	n	y	GS ≥ 3 + 4
		2	Apr 2013–Oct 2016		251							
Niu et al. [25]	2017	1	Jan 2014–Sep 2015	+PSA ± DRE ± RUS	151	mpMRI	n.r.	n.r.	12-cores TR	n	y	GS ≥ 7
		1	Oct 2015–Oct 2016		74					n	y	GS ≥ 7
Radtke et al. [21]	2017	1	2012–2015	+PSA ± DRE	660	mpMRI	P2-5	fusion	24-cores TP	n	y	GS ≥ 3 + 4
**Prior negative biopsy setting**												
Alberts et al. [20]	2018	5	2012–2015	+PSA ± DRE, persistant suspicion	504	mpMRI	P3-5	Fusion	12-cores TR	y	y	GS ≥ 3 + 4
Bjurlin et al. [30] (Eur Urol Focus)	2018	1	Apr 2012–Sept 2017	+PSA ± DRE, persistant suspicion	104	mpMRI	P3-5	Fusion	12-cores TR	y	n	
Bjurlin et al. [24] (Urology)	2018	1	Jun 2012–Aug 2014	+PSA ± DRE, persistant suspicion	119	mpMRI	P3-5	Fusion	12-cores TR	y	y	GS ≥ 7
		1	Jun 2012–Aug 2014		52							
Huang et al. [27]	2018	1	Jan 2007–Apr 2017	+PSA ± DRE, persistant suspicion	231	mpMRI	P3-5	n.r.	12-cores TR	y	y	GS ≥3+4
Radtke et al. [21]	2017	1	2012–2017	+PSA ± DRE, persistant suspicion	355	mpMRI	P2-5	fusion	24-cores TP	n	y	GS ≥ 3 + 4
Truong [26]	2018	2	Dec 2014–Dec 2016; Feb 2014–Feb 2017	+PSA ± DRE, persistant suspicion	285	mpMRI	P3-5	Fusion	12-cores TR	y	n	n.a.
**Combined biopsy-naive and prior negative biopsy setting**												
Lee et al. [28]	2017	1	Jul 2012–Nov 2015	+PSA ± DRE, persistant suspicion	615	bpMRI	P3-5	Fusion	24-40 cores TP	y	y	GS ≥ 3 + 4 /MCCL ≥ 6mm
Van Leeuwen et al. [29]	2018	1	Apr 2012–Mar 2014	+PSA ± DRE, persistant suspicion	393	mpMRI	P3-5	Fusion/cognitive	30-cores TP	n	y	GS ≥ 3 + 4 (> 5%)/MCCL ≥ 7 mm/ ≥ 20% core+
		1	Jan 2013–Dec 2014		198	mpMRI	P3-5	Cognitive	18-cores TR/TP

**Table 2:** Characteristics and results of studies related to nomogram development for prostate cancer diagnosis, including multi-parametric MRI

Study		Nomogram														Results	
		Developing/validation cohort	Input parameters baseline model								Additional input parameters MRI-model			Prevalence		C-statistic (area under the ROC curve)	
Patient groups	Year	Developing/validation cohort	Age	PSA	PSAD	Prostate volume	DRE	TRUS	Etnicity	Prior neg. bx	MRI suspicion score	Prostate volume (MRI)	Age	All PCa	csPCa	all PCa	csPCa
**Biopsy-naive setting**																	
Alberts et al. [20]	2018	Developing	n	y	n	y	y	y	n	n	y	n	y	58.40%	42.30%	0.84	0.84
Bjurlin et al. [24]	2018	Developing	y	n	y	n	n	n	n	n	y	n	n	55.20%	34.80%	0.82	0.91
		Validation												47.10%	31.00%	0.78	0.84
Fang et al. [23]	2016	Developing	y	y	n	y	y	y	n	n	y	n	n	48.50%	24.40%	0.88	0.87
Mehralivand et al. [22]	2018	Developing	y	y	n	n	y	n	y	y	y	y	n	n.r.	48.30%	n.a.	0.84
		Validation												n.r.	38.20%	n.a.	0.84
Niu et al. [25]	2017	Developing	y	n	y (MRI)	n	n	n	n	n	y	n	n	56%	21%	n.a.	0.85
		Validation												52%	24%	n.a.	0.82
Radtke et al. [21]	2017	Developing and (internally) validation	y	y	n	y	y	n	n	n	y	n	n	n.r.	45.80%	n.a.	0.84
**Prior negative biopsy setting**																	
Alberts et al. [20]	2018	Developing	n	y	n	y	y	y	n	y	y	n	y	43.10%	28.90%	0.79	0.85
Bjurlin et al. [30]	2018	Validation	y	y	n	y	n	n	n	y	y	n	n	70.20%	49.00%	0.78	..
Bjurlin et al. [24]	2018	Develop	y	n	y	n	n	n		n	y	n	n	31.90%	21.80%	0.76	0.86
		Validation												28.80%	9.60%	0.65	0.87
Huang et al. [27]	2018	Developing	y	y	n	y	y	n	n	n	y	n	y	32.50%	25.50%	0.88	0.93
Radtke et al. [21]	2017	Developing and (internally) validation	y	y	n	y	y	n	n	n	y	n	n	n.r.	39.80%	n.a.	0.78
Truong [26]	2018	Developing and (internally) validation	y	y	n	y	n	n	n	y	y	n	n	53.70%	29.00%	0.83	n.a.
**Combined biopsy-naive and prior negative biopsy setting**																	
Lee et al. [28]	2017	Developing and (internally) validation	y	n	y (MRI)	n	n	n	n	y	y	n	n	51.50%	38.50%	0.87	0.92
Van Leeuwen et al. [29]	2018	Developing	y	y	n	y	y	n	n	y	y	n	n	57.70%	37.90%	n.a.	0.88 / 0.90
		Validation												71.10%	44.70%	n.a.	0.86

### In biopsy-naïve setting

Several studies have developed an MRI risk prediction model, which some have been internally or externally validated.

Based on the ERSPC-RCs, a risk calculator including mpMRI was developed on datasets from five international centers [20]. Input variables were PSA, DRE, prostate volume, prior biopsies, age, and mpMRI (PI-RADS 1–5) (Table 2). In total, data from 504 biopsy-naïve men were used to adjust the ERSPC-RC3 into the MRI-ERSPC-RC3. MRI-ERSPC-RC3 had a significantly higher AUC for *G* ≥ 2 PCa compared with the non-calibrated baseline model (ERSPC-RC3 + DRE): 0.84 [95% confidence interval (CI) 0.81–0.88] versus 0.76 [0.71–0.80] (*p* < 0.01) (Table 2). In decision curve analysis, the MRI-ERSPC-RC3 showed a benefit for G ≥ 2 PCa threshold probabilities larger than 10%. For example, at a risk threshold of ≥ 10% *G* ≥ 2 PCa to indicate a biopsy, 14% (143/1000) biopsies would have been avoided, missing *G* = 1 PCa in 13% (18/143) and missing *G* ≥ 2 PCa in 10% (14/143) of the avoided biopsies (i.e., negative test) (Table 3). We need to address that the prevalence of 42% *G* ≥ 2 PCa in this cohort was rather high. At this threshold, still only 3.3% of all *G* ≥ 2 PCa (14/423) was not detected overall. Due to this high-risk population, hardly any benefit was reached at a risk threshold of lower than 10% *G* ≥ 2 PCa.

**Table 3:** Test results of baseline model and MRI-model in biopsy-naive, prior negative biopsy, and combined setting

Study	Test threshold	Baseline-model								MRI-model							
		*Test results percentage*								*Test results percentage*							
		Positive test			Negative test					Positive test			Negative test				
		Men biopsied	csPCa detected	cisPCa detected	Men not biopsied	csPCa missed	cisPCa missed	csPCa missed	cisPCa missed	Men biopsied	csPCa detected	cisPCa detected	Men not biopsied	csPCa missed	cisPCa missed	csPCa missed	cisPCa missed
		(% of all men)	TP (% of A)	(% of A)	(% of all men)	FN (% of B)	(% of B)	(% of all csPCa)	(% of all cisPCa)	(% of all men)	TP (% of A)	(% of A)	(% of all men)	FN (% of B)	(% of B)	(% of all csPCa)	(% of all cisPCa)
**Biopsy-naive setting**																	
Alberts et al. [20]	0%	100.0%	42.3%	16.1%	0.0%	0.0%	0.0%	0.0%	0.0%	100.0%	42.3%	16.1%	0.0%	0.0%	0.0%	0.0%	0.0%
	5%	99.8%	42.4%	16.1%	0.2%	0.0%	0.0%	0.0%	0.0%	98.0%	42.9%	16.2%	2.0%	15.0%	10.0%	0.7%	1.2%
	10%	99.4%	42.4%	16.2%	0.6%	33.3%	0.0%	0.5%	0.0%	85.7%	47.7%	16.7%	14.3%	9.8%	12.6%	3.3%	11.2%
	15%	94.6%	43.4%	16.2%	5.4%	22.2%	14.8%	2.8%	5.0%	76.2%	52.9%	17.7%	23.8%	8.4%	10.9%	4.7%	16.1%
	20%	84.3%	47.6%	17.2%	15.7%	14.0%	10.2%	5.2%	9.9%	67.5%	57.6%	16.1%	32.5%	10.5%	16.0%	8.0%	32.3%
Fang et al. [23]	0%	100.0%	24.4%	24.1%	0.0%	0.0%		0.0%		100.0%	24.4%	24.1%	0.0%	0.0%	0.0%	0.0%	
	5%																
	10%	90.1%	27.0%		9.9%	1.0%		0.2%		90.2%	27.1%		9.8%	0.0%		0.0%	
	15%																
	20%	80.2%	29.6%		19.8%	3.5%		1.7%		80.2%	30.0%		19.8%	1.5%		0.7%	
Mehralivand et al. [22]	0%	100.0%	38.2%	19.8%	0.0%	0.0%		0.0%		100.0%	38.2%	19.8%	0.0%	0.0%	0.0%	0.0%	
	5%																
	10%	100.0%	38.2%		0.0%	0.0%		0.0%		82.8%	44.8%		17.2%	6.4%		2.9%	
	15%	98.1%	38.9%		1.9%	0.0%		0.0%		75.0%	48.9%		25.0%	6.1%		4.0%	
	20%	94.7%	39.9%		5.3%	7.5%		1.0%		62.4%	54.5%		37.6%	11.2%		11.0%	
Radtke et al. [21]	0%	100.0%	45.8%		0.0%	0.0%		0.0%		100.0%	45.8%		0.0%	0.0%	0.0%	0.0%	0.0%
	5%	97.5%	46.1%		2.5%	36.0%		2.0%		98.9%	46.3%		1.1%	0.0%		0.0%	
	10%	94.7%	47.4%		5.3%	17.0%		2.0%		89.9%	49.9%		10.1%	8.9%		2.0%	
	15%																
	20%	82.1%	52.5%		17.9%	15.1%		5.9%		71.9%	58.6%		28.1%	13.2%		8.1%	
**Prior negative biopsy setting**																	
Alberts et al. [20]	0%	100.0%	28.9%	14.2%	0.0%	0.0%	0.0%	0.0%	0.0%	100.0%	28.9%	14.2%	0.0%	0.0%	0.0%	0.0%	0.0%
	5%	99.7%	29.0%	14.2%	0.3%	0.0%	0.0%	0.0%	0.0%	73.5%	38.4%	14.3%	26.5%	2.6%	14.0%	2.4%	26.1%
	10%	90.1%	31.1%	13.5%	9.9%	9.1%	20.2%	3.1%	14.1%	63.9%	42.9%	13.6%	36.1%	4.2%	15.2%	5.2%	38.7%
	15%	72.0%	37.1%	15.6%	28.0%	7.9%	10.7%	7.6%	21.1%	58.6%	45.6%	14.5%	41.4%	5.3%	13.8%	7.6%	40.1%
	20%	60.2%	39.7%	14.0%	39.8%	12.6%	14.6%	17.3%	40.8%	52.5%	48.8%	14.5%	47.5%	6.9%	13.9%	11.4%	46.5%
Radtke et al. [21]	0%	100.0%	39.8%		0.0%	0.0%		0.0%		100.0%	39.8%		0.0%	0.0%		0.0%	
	5%	98.0%	39.8%		2.0%	40.0%		2.0%		97.6%	40.8%		2.4%	0.0%		0.0%	
	10%	93.6%	40.8%		6.4%	25.0%		4.0%		89.2%	43.3%		10.8%	11.1%		3.0%	
	15%																
	20%	80.6%	45.4%		19.4%	16.5%		8.0%		68.7%	50.9%		31.3%	15.3%		12.1%	
**Combined biopsy-naive and prior negative biopsy setting**																	
Lee et al. [28]	0%	100.0%	38.5%	13.0%						100.0%	38.5%	13.0%	0.0%	0.0%	0.0%	0.0%	0.0%
	5%									95.6%	40.1%	13.1%	4.4%	3.7%	11.1%	0.4%	3.8%
	10%									89.4%	42.7%	12.9%	10.6%	3.1%	13.8%	0.8%	11.3%
	15%									82.0%	46.4%	12.5%	18.0%	2.7%	15.3%	1.3%	21.3%
	20%									74.1%	51.0%	12.5%	25.9%	2.5%	14.5%	1.7%	28.8%
Van Leeuwen et al. [29]	0%	100.0%	37.9%	57.8%	0.0%	0.0%	0.0%	0.0%	0.0%	100.0%	37.9%	57.8%	0.0%	0.0%	0.0%	0.0%	0.0%
	5%	92.6%	40.7%	61.0%	7.4%	2.7%	17.6%	0.5%	2.2%	81.4%	46.6%	66.2%	18.6%	0.0%	20.4%	0.0%	6.6%
	10%	84.5%	44.3%	64.5%	15.5%	3.2%	21.3%	1.3%	5.7%	71.8%	51.4%	70.2%	28.2%	3.5%	26.2%	2.6%	12.8%
	15%	74.6%	48.8%	67.8%	25.4%	5.9%	28.3%	4.0%	12.5%	62.1%	56.5%	74.6%	37.9%	7.4%	30.3%	7.4%	19.9%
	20%																

Radtke and coworkers [21] investigated multivariate prediction modeling in a similar approach, also based on the ERSPC-RC3. A single-center dataset of 660 biopsy-naïve men was used. MRI-ERSPC-RC3 reached a higher AUC (0.83), compared with ERSPC-RC3 (0.81), age refitted ERSPC-RC3 (0.80, *p* < 0.001), and PI-RADSv1.0 (0.76, *p* < 0.001) (Table 2). In decision curve analysis, the MRI-ERSPC-RC3 showed a benefit for *G* ≥ 2 PCa threshold probabilities larger than 10% (Table 3). Also in this study cohort, the estimated prevalence for *G* ≥ 2 PCa was 46% and was rather high, explaining the benefit in only the risk threshold above 10%.

Mehralivand and colleagues developed a risk prediction model, based on PSA, DRE, age, African American ethnicity, and incorporating mpMRI (PI-RADS 1–5) [22]. A developing cohort from 1 institute (*n* = 400) and a validating cohort from 2 other institutes (*n* = 250) had prevalence for *G* ≥ 2 PCa (outcome measurement) of 48.3% and 38.2%, respectively. In comparison to the baseline model, the AUC of the MRI risk prediction model increased from 0.72 to 0.84 (*p* < 0.001) in the development cohort, and increased from 0.64 to 0.84 (*p* < 0.001) in the validation cohort (Table 2). By applying the MRI risk prediction model to the validation cohort, higher net benefit than the baseline model could be achieved for risk thresholds above 10%. At a risk threshold of ≥ 10% *G* ≥ 2 PCa to indicate a biopsy, 17% (172/1000) biopsies would have been avoided, missing *G* ≥ 2 PCa in 6% (11/172) in the avoided biopsies (Table 3). In this study cohort, the prevalence for *G* ≥ 2 PCa was 38% and was a little lower than in the previous studies, which may contribute to the higher net benefit at the same risk threshold of ≥ 10%.

Fang et al. [23] developed an MRI risk prediction model, based on PSA, age, and (abnormal) transrectal ultrasound, incorporating mpMRI (PI-RADS 1–5). The AUC for *G *≥ *3* PCa for the developing cohort (*n* = 894, with a prevalence 24.4%) was 0.87, in comparison to 0.85 (*p* = 0.001) risk prediction model without mpMRI (Table 2). At a risk threshold of ≥ 10% *G* ≥ 3 PCa to indicate a biopsy, 10% (98/1000) biopsies would have been avoided, missing no *G* ≥ 3 PCa (0%, 0/98) in the avoided biopsies (Table 3).

Bjurlin and colleagues [24] developed an MRI risk prediction model, based on PSA-density and age, incorporating mpMRI (MRI suspicion score assessment category 3–5, excluding the first categories). Bias-corrected AUC for *G* ≥ 2 (Gleason ≥ 7) PCa was 0.91 for the developing cohort (*n* = 201, with a prevalence 34.8%) and 0.84 for the validating cohort (*n* = 87, with a prevalence 31.0%) (Table 2). These AUCs were higher in comparison to baseline model (PSA-density) (0.75 and 0.69) and MRI suspicion score model (0.90 and 0.84).

Niu and coworkers [25] developed an MRI risk prediction model, based on adjusted PSA-density and age, incorporating mpMRI (PI-RADS 1–5). The AUC for *G* ≥ 2 (Gleason score ≥ 7) for the developing and validating cohort was 0.85 [0.79–0.90] (*n* = 151, with a prevalence 21.2%) and 0.82 [0.76–0.89] (*n* = 74, with a prevalence 24.3%) (Table 2). These AUCs were higher in comparison to the baseline model (PSA-density) (0.74 [0.66–0.79], *p* = 0.013) and PI-RADSv2 score (0.76 [0.71–0.84], *p* = 0.018).

### In prior negative biopsy setting

Next to the development of MRI-ERSPC-RC3 on biopsy-naïve men, a risk calculator MRI-ERSPC-RC4 was developed for men with prior negative biopsy (*n* = 457, prevalence of 29% *G* ≥ 2 PCa) [20]. MRI-ERSPC-RC4 had a significantly higher AUC for *G* ≥ 2 PCa compared with the ERSPC-RC4 + DRE (baseline model): 0.85 (95% CI 0.81–0.89) versus 0.74 (95% CI 0.69–0.79, *p* < 0.01) (Table 2). Using a ≥ 10% risk threshold for *G* ≥ 2 PCa to indicate a biopsy, 36% (361/1000) biopsies would have been avoided, missing *G* = 1 PCa in 15% (55/361) and missing *G* ≥ 2 PCa in 4% (15/361) of all avoided biopsies (Table 3). In this cohort the prevalence of 29% *G* ≥ 2 PCa was lower than for the biopsy-naïve cohort. Therefore, in contrast to biopsy-naïve men, the decision curve analysis in men with prior negative biopsy already showed clear net benefit of the MRI-ERSPC-RC4 at a risk threshold of ≥ 5% for *G* ≥ 2 PCa (Table 3).

Radtke and coworkers [21] also developed an MRI-ERSPC-RC4 for men with prior negative biopsy, next to an MRI-ERSPC-RC3. In men with previous biopsy (*n* = 355, estimated prevalence of 40% *G* ≥ 2 PCa), the discrimination of the MRI-ERSPC-RC4 (0.81) was superior to that of ERSPC-RC4 (baseline model) (0.66, *p* < 0.001), refitted ERSPC-RC4 (0.76, *p* < 0.001), and PI-RADSv1.0 (0.78, *p* < 0.001) (Table 2). In decision curve analysis, the MRI-ERSPC-RC4 showed a benefit for *G* ≥ 2 PCa threshold probabilities larger than ≥ 10% (Table 3).

Bjurlin and colleagues [24] investigated an MRI risk prediction model with a bias-corrected AUC for *G* ≥ 2 PCa of 0.86 for the developing cohort (*n* = 119, with a prevalence 21.8% *G* ≥ 2 PCa) and 0.87 for the validating cohort (*n* = 52, with a prevalence 9.6% *G* ≥ 2 PCa) (Table 2). These AUCs were higher in comparison to the baseline model (PSA-density) (0.76 and 0.76), and MRI suspicion score model (0.83 and 0.84).

Truong et al. [26] developed an MRI risk prediction model, based on age, PSA, and prostate volume, incorporating mpMRI (PI-RADS 1–5). The AUC for predicting *benign* prostate pathology for the developing cohort (*n* = 285, with a prevalence 46.3% benign prostate) was 0.83 (Table 2). With a cutoff probability of ≥ 0.70 used to recommend deferment of MRI-targeted biopsy, in 21.4% (61/285) men unnecessary biopsy would have been avoided, and 6.2% (4/61) with a *G* ≥ 2 PCa would have been missed. The prevalence of *G* ≥ 2 PCa in this cohort was 39% (111/285). Overall, only 3.6% (4/111) of all *G* ≥ 2 PCa would not have been detected at this threshold. Another group [30] validated this prediction tool, and found for predicting benign prostate pathology an AUC of 0.78. An updated model was constructed with improved calibration and similar discrimination (AUC 0.79).

Huang et al. [27] developed an MRI risk prediction model, based on age, PSA, prostate volume, and DRE, incorporating mpMRI (PI-RADS 1–5). The AUC for *G* ≥ 2 PCa for the developing cohort (*n* = 231, with a prevalence 25.5%) was 0.93 [0.89–0.96] (*p *< 0.001) (Table 2).

### In combined biopsy-naïve and prior negative biopsy setting

Lee et al. [28] developed an MRI risk prediction model, based on age, PSA-density, and primary biopsy, incorporating biparametric MRI (PI-RADS 1–5). The AUC for csPCa (*G* ≥ 2 or MCCL ≥ 6 mm) for the developing cohort (*n* = 615, with a prevalence 38.5%) was 0.92 [0.89–0.94] (Table 2). At a calculated probability cutoff of ≥ 10% csPCa to indicate a biopsy, 10.6% (65/615) biopsies would have been avoided, missing *G* = 1 PCa in 16.9% (11/65) and missing G ≥ 2 PCa in 3.1% (2/65) of the avoided biopsies (Table 3).

Van Leeuwen and coworkers [29] developed a risk prediction model, based on age, PSA, DRE, prostate volume, previous biopsy, incorporating mpMRI (MRI suspicion score category 1–5). csPCa was defined as Gleason 7 with > 5% grade 4, ≥ 20% cores positive or ≥ 7 mm maximum cancer core length (MCCL). The AUC for csPCa was 0.88 [0.85–0.92] for the developing cohort (*n* = 393, with a prevalence 37.9%) and 0.86 [0.81–0.92] for the validating cohort (*n* = 198, with a prevalence 44.7%) (Table 2). This was higher than in comparison to the multivariate risk baseline model (0.80 [0.75–0.84]). With a cutoff probability of ≥ 0.10 used to indicate a biopsy, in 28.2% (282/1000) men an unnecessary biopsy would have been avoided, missing *G* = 1 PCa in 26.2% (74/282) and missing *G* ≥ 2 PCa in 3.5% (10/282) in the avoided biopsies. Overall, 12.8% (74/578) cisPCa and 2.6% (10/379) csPCa were not detected at this threshold.

## Performance analysis of the MRI risk prediction models

Next to the comparison of AUCs between MRI-models, the performance can also be further explored using other performance parameters (Table 4). For such an analysis, performance parameters like true positive rate (TPR), false positive rate (FPR), net benefit (NB), percentage avoided biopsies (PAB), and percentage avoided clinically insignificant PCa could be investigated, as shown by Mehralivand and coworkers [22]. We were able to calculate these performance parameters for most studies, with risk thresholds ranging from 0 to 20% (Table 4).

**Table 4:** Performance measures related to clinical usefulness of baseline model, MRI-model, and net differences between both models, in biopsy-naive, prior negative biopsy, and combined setting

Study	Test threshold	Baseline-model					MRI-model					Comparison MRI- and baseline-model				
		Performance measures related to clinical usefulness					Performance measures related to clinical usefulness					Performance measures related to clinical usefulness				
		Avoided Biopsy	Avoided cisPCa	True positive rate (TPR) Sensitivity	False positive rate (FPR) 1-Specificity	Net benefit (NB)	Avoided Biopsy	Avoided cisPCa	True positive rate (TPR) Sensitivity	False positive rate (FPR) 1-Specificity	Net benefit (NB)	Avoided Biopsy	Avoided cisPCa	True positive rate (TPR) Sensitivity	False positive rate (FPR) 1-Specificity	Net benefit (NB)
**Biopsy-naive setting**																
Alberts et al. [20]	0%	0.000	0.000	1.000	1.000	0.000	0.000	0.000	1.000	1.000	0.000	0.0%	0.0%	0.0%	0.0%	0.0%
	5%	0.002	0.000	1.000	0.997	0.393	0.020	0.012	0.993	0.971	0.391	1.8%	1.2%	− 0.7%	− 2.6%	− 0.2%
	10%	0.006	0.000	0.995	0.993	0.357	0.143	0.112	0.967	0.776	0.359	13.7%	11.2%	− 2.8%	− 21.7%	0.2%
	15%	0.054	0.050	0.972	0.927	0.317	0.238	0.161	0.953	0.622	0.340	18.4%	11.2%	-1.9%	-30.5%	2.3%
	20%	0.157	0.099	0.948	0.766	0.291	0.325	0.323	0.920	0.496	0.318	16.8%	22.4%	− 2.8%	− 27.0%	2.7%
Fang et al. [23]	0%	0.000		1.000	1.000	0.000	0.000		1.000	1.000	0.000	0.0%		0.0%	0.0%	0.0%
	5%															
	10%	0.099		0.995	0.871	0.170	0.098		1.000	0.870	0.171	− 0.1%		0.5%	− 0.1%	0.1%
	15%															
	20%	0.198		0.972	0.747	0.096	0.198		0.986	0.743	0.100	0.0%		1.4%	− 0.4%	0.4%
Mehralivand et al. [22]	0%	0.000		1.000	1.000	0.000	0.000		1.000	1.000	0.000	0.0%		0.0%	0.0%	0.0%
	5%															
	10%	0		1.000	1.000	0.313	0.172		0.970	0.739	0.320	17.2%		− 3.0%	− 26.1%	0.7%
	15%	0.019		1.000	0.969	0.276	0.250		0.960	0.620	0.299	23.1%		− 4.0%	− 34.9%	2.3%
	20%	0.053		0.990	0.921	0.236	0.376		0.890	0.460	0.269	32.3%		− 10.0%	− 46.1%	3.3%
Radtke et al. [21]	0%	0.000		1.000	1.000	0.000	0.000		1.000	1.000	0.000	0.0%		0.0%	0.0%	0.0%
	5%	0.025		0.980	0.970	0.421	0.011		1.000	0.980	0.430	− 1.4%		2.0%	0.9%	0.9%
	10%	0.053		0.980	0.919	0.394	0.101		0.980	0.830	0.399	4.8%		0.0%	− 8.9%	0.5%
	15%															
	20%	0.179		0.941	0.720	0.334	0.281		0.919	0.550	0.347	10.2%		− 2.2%	− 17.0%	1.3%
**Prior negative biopsy setting**																
Alberts et al. [20]	0%	0.000	0.000	1.000	1.000	0.000	0.000	0.000	1.000	1.000	0.000	0.0%	0.0%	0.0%	0.0%	0.0%
	5%	0.003	0.000	1.000	0.996	0.252	0.265	0.261	0.976	0.637	0.258	26.2%	26.1%	− 2.4%	− 35.9%	0.6%
	10%	0.099	0.141	0.969	0.873	0.211	0.361	0.387	0.948	0.513	0.233	26.2%	24.6%	− 2.1%	− 36.0%	2.2%
	15%	0.28	0.211	0.924	0.637	0.187	0.414	0.401	0.924	0.449	0.211	13.4%	19.0%	0.0%	− 18.8%	2.4%
	20%	0.398	0.408	0.827	0.511	0.148	0.475	0.465	0.886	0.378	0.189	7.7%	5.6%	5.9%	− 13.2%	4.1%
Radtke et al. [21]	0%	0.000		1.000	1.000	0.000	0.000		1.000	1.000	0.000	0.0%		0.0%	0.0%	0.0%
	5%	0.020		0.980	0.980	0.359	0.024		1.000	0.960	0.368	0.4%		2.0%	− 2.0%	0.9%
	10%	0.064		0.960	0.920	0.320	0.108		0.970	0.841	0.330	4.4%		1.0%	− 8.0%	0.9%
	15%															
	20%	0.194		0.920	0.731	0.256	0.313		0.879	0.560	0.266	11.9%		− 4.0%	− 17.1%	1.0%
**Combined biopsy-naive and prior negative biopsy setting**																
Lee et al. [28]	0%	0.000		1.000	1.000	0.000	0.000	0.000	1.000	1.000	0.000	0.0%		0.0%	0.0%	0.0%
	5%						0.044	0.038	0.996	0.931	0.353					
	10%						0.106	0.113	0.992	0.833	0.325					
	15%						0.180	0.213	0.987	0.714	0.303					
	20%						0.259	0.288	0.983	0.590	0.288					
Van Leeuwen et al. [29]	0%	0.000	0.000	1.000	1.000	0.000	0.000	0.000	1.000	1.000	0.000	0.0%	0.0%	0.0%	0.0%	0.0%
	5%	0.074	0.022	0.995	0.884	0.348	0.186	0.066	1.000	0.700	0.356	11.2%	4.3%	0.5%	− 18.4%	0.8%
	10%	0.155	0.057	0.987	0.758	0.322	0.282	0.128	0.974	0.562	0.330	12.7%	7.1%	− 1.3%	− 19.6%	0.9%
	15%	0.254	0.125	0.960	0.615	0.297	0.379	0.199	0.926	0.435	0.303	12.5%	7.4%	− 3.4%	− 18.0%	0.7%
	20%															

By analyzing the individual performance parameters, physician and patient may be better informed to decide whether to undergo further biopsy testing or no further biopsy testing. A better trade-off can be made, based on the subjective weight of each individual performance parameter. If a patient focuses on maximizing diagnostic yield, the TPR is of vital importance in his decision. If a patient focuses on not to be (unnecessarily) biopsied, the FPR and PAB become more prominent. The net benefit is calculated by the formula = (TP − *w* FP)/*N*), where TP is the number of true positive decisions, FP the number of false positive decisions, *N* is the total number of patients and *w* is a weight equal to the odds of the risk threshold or cutoff in percentages (pt/(1 − pt) [31].

In most studies, the MRI risk prediction models were compared to baseline risk prediction models (without MRI). The input parameters in the used baseline models differed between studies, as previously discussed. The comparison between the each MRI- and baseline model is in fact the calculated difference between the values of the individual performance parameter (Table 4). For example, using the MRI-model instead of the baseline model in biopsy-naïve men at a risk threshold of ≥ 10%, Alberts and coworkers showed a net difference of 13.7% in avoiding biopsies, 11.2% in avoiding the detection of clinically insignificant PCa, 21.7% reduction of false positive tests, at the expense of 2.8% not detected csPCa.

Using the MRI-risk model instead of the baseline risk model in biopsy-naïve men, we observe that three studies show a notable beneficial net difference above the threshold of > 10%, at least for the FPR, NB, avoided biopsies and avoided detection of clinically insignificant PCa [20–22]. These results are at a low price of missing csPCa, even in the investigated cohorts with remarkable high prevalence of clinically significant PCa (42.3–48.3%) [20–22] (Table 2).

These results would have been even better in more real-life opportunistic screening cohorts, instead of the highly selected cohorts in these tertiary hospitals. Following upfront risk prediction in biopsy-naïve men, the prevalence of csPCa might be in the range of approximately 20–30% [13, 32]. Before using such a test recalibration of these multivariate risk prediction tools to the in-hospital diagnosed population is mandatory.

## Considerations and future perspectives

Individualized risk assessment of csPCa using a predictive model that incorporates the mpMRI suspicion score in combination with clinical and biochemical data, allows a considerable reduction in unnecessary biopsies and reduction of the risk of over-detection of clinically insignificant PCa, albeit at the expense of a low number of (not diagnosed) csPCa. Whether or not this has detrimental effect on future metastatic and mortality rates remains to be seen. The MRI risk prediction models highlight their accuracy and power, and suggest that the usage of these tools would allow the identification of patients with significant disease. However, at present external validation and results of calibrating steps are lacking.

We need to acknowledge that previously developed and already validated risk calculators, without mpMRI as an input variable, perform sufficient, but can be further improved by the input of other “biomarkers” such as mpMRI. Despite these satisfactory predictive results and potential improvements in outcomes, MRI risk prediction models may also avoid limitations of these models. For example, prostate volume is an important input variable as it is related to PSA value. Prostate volume is commonly determined on TRUS or in certain circumstances estimated by DRE. The use of a TRUS-based input variable, including the variable of abnormal or suspicious TRUS, is impractical since men who undergo TRUS are also likely to undergo TRUS-guided biopsy. With mpMRI, prostate volume can also be accurately assessed, and avoiding the need for performing a TRUS [33, 34]. It provides information on probably the most strong predictor for biopsy outcome currently available, i.e., PSA-density. Therefore, the results of the MRI-based models can be used to counsel men in biopsy decision-making, even before planning a TRUS. Still, the indication for prostate MRI should be critically evaluated, when even unnecessary mpMRI’s can be avoided by multivariate risk prediction [35, 36]. In such a scenario, at first the mpMRI could be circumvented using DRE to the roughly estimate prostate volume. Subsequently, mpMRI could only be utilized in men that most likely have an elevated PSA not related to BPH [37–41].

Despite the good performance of these constructed and published MRI risk prediction models, in combination with the potential advantages, we need to address some limitations for its generalizability and practicality in daily clinic.

The accuracy of the systematic biopsy is dependent on the number of cores. Most studies reported a transrectal approach with a median of 12 biopsy cores (Table 1) [20, 22–27, 30]. However, three studies used a transperineal approach with biopsy cores ranging from 24 to 40 [21, 28, 29]. This is important to realize when such a model is applied in a setting where this biopsy technique is not implemented as a routine daily based practice. Furthermore, the results of biopsy schemes involving saturation biopsies appear to have a higher concordance rate with results from prostatectomy than a scheme involving 12 cores, indicating that these tools predicts more accurate the risk of csPCa [42]. However, in a literature review the increase in diagnostic yield becomes marginal as the number of cores increases above 12 [42, 43]. Furthermore, some existing risk calculators are derived from a six or eight core approach, in which the reference test may be less reliable [44, 45]. Moreover, different targeting methods (MRI/US fusion or cognitive biopsy) were used. Subsequently, when systematic biopsies are performed with the knowledge of the mpMRI findings, the results from this input variable might overestimate the diagnostic performances of the multivariable model.

Next to prevalence of csPCa, the characteristics of the population tested, may influence the outcome of the models. Therefore, again, the applicability of the predicted risk should match with the risk in the investigated population. For example, higher PSA value combined with a higher positive rate of DRE or abnormal TRUS findings, may select a population with a high-risk for csPCa, and may reduce the additional value of mpMRI. Moreover, when experienced radiologists have interpreted the mpMRIs in the cohorts used for the predictive models, the results of these models may not be applicable to less experienced radiologists.

The constructed MRI risk prediction models were based on retrospective data with a probable risk of selection bias. Furthermore, these models were constructed with relatively small sample sizes, mostly from a single institution that may hamper the extrapolation and interpretation into larger cohorts.

Internal validation was performed on an even smaller (single-center) sample size and AUCs dropped in some examples [24, 25, 29]. Prediction tools require external validation in a multicenter study to assess their wider applicability. Only one study externally validated a constructed MRI risk model [30] with a small drop in AUC as compared to the development cohort [26].

Novel biomarkers for PCa with prognostic value will be developed. Ancillary tests such as the prostate cancer antigen 3 (PCA3) test, prostate health index, 4Kscore test, and ConfirmMDx may also be considered to provide further reassurance about omitting prostate biopsy. As a next step, these markers will be integrated in the multivariate risk prediction tools. One study reported the analysis of PCA3 in their report; however, in the multivariate analysis PCA3 did not sustain significance as an independent predictor [24].

Above all, we should take care investigating these MRI-based risk prediction models and make sure that in our surge of enthusiasm we do not throw the baby out with the bathwater. We do have already validated risk prediction models with satisfactory performance [13–19], however, the utility of these models are not completely adopted in daily practice, despite recommendation by international guidelines [3, 4]. Further improvement in selecting only those men who will benefit from mpMRI will be essential in the near future. The additional value of mpMRI in risk prediction on top off current models should be carefully analyzed. These new MRI risk assessment tools require external validation on different patient populations who possess varying baseline risks to ensure that the risk prediction tool performs satisfactorily prior to implementation in clinical practice.

## Conclusions

Due to the predictive value of mpMRI in prostate cancer diagnosis, recently new multivariate risk prediction tools have been constructed, with the inclusion of the mpMRI suspicion score. All MRI risk prediction models had a high accuracy with area’s under the receiver-operating characteristic curves ranging from 0.78 to 0.93, and suggest that the usage of these tools would allow the selective identification of patients with significant disease. By analyzing individual performance parameters, physician and patient may be better informed to decide whether or not to undergo further biopsy testing.
